# Molecular-based cross-species evaluation of bovine coronavirus infection in cattle, sheep and goats in Ghana

**DOI:** 10.1186/s12917-020-02606-x

**Published:** 2020-10-27

**Authors:** Vitus Burimuah, Augustina Sylverken, Michael Owusu, Philip El-Duah, Richmond Yeboah, Jones Lamptey, Yaw Oppong Frimpong, Olivia Agbenyega, Raphael Folitse, Ben Emikpe, William Tasiame, Eddie-Williams Owiredu, Samuel Oppong, Christopher Antwi, Yaw Adu-Sarkodie, Christian Drosten

**Affiliations:** 1 Department of Clinical Microbiology, Kwame Nkrumah University of Science and Technology, Kumasi, Ghana; 2 School of Veterinary Medicine, Kwame Nkrumah University of Science and Technology, Kumasi, Ghana; 3 Kumasi Centre for Collaborative Research in Tropical Medicine, Kumasi, Ghana; 4 Department of Theoretical and Applied Biology, Kwame Nkrumah University of Science and Technology, Kumasi, Ghana; 5 Department of Medical Laboratory Technology, Kwame Nkrumah University of Science and Technology, Kumasi, Ghana; 6 Institute of Virology, Universitätsmedizin Berlin, Charite, Germany; 7 Department of Animal Science, Kwame Nkrumah University of Science and Technology, Kumasi, Ghana; 8 Department of Agroforestry, Kwame Nkrumah University of Science and Technology, Kumasi, Ghana; 9 Department of Molecular Medicine, Kwame Nkrumah University of Science and Technology, Kumasi, Ghana; 10 Department of Wildlife and Range Management, Kwame Nkrumah University of Science and Technology, Kumasi, Ghana

**Keywords:** Bovine coronavirus, Cattle, Sheep, Goat, Cross-species infection

## Abstract

**Background:**

Apart from the huge worldwide economic losses often occasioned by bovine coronavirus (BCoV) to the livestock industry, particularly with respect to cattle rearing, continuous surveillance of the virus in cattle and small ruminants is essential in monitoring variations in the virus that could enhance host switching. In this study, we collected rectal swabs from a total of 1,498 cattle, sheep and goats. BCoV detection was based on reverse transcriptase polymerase chain reaction. Sanger sequencing of the partial RNA-dependent RNA polymerase (RdRp) region for postive samples were done and nucleotide sequences were compared with homologous sequences from the GenBank.

**Results:**

The study reports a BCoV prevalence of 0.3%, consisting of 4 positive cases; 3 goats and 1 cattle. Less than 10% of all the animals sampled showed clinical signs such as diarrhea and respiratory distress except for high temperature which occurred in > 1000 of the animals. However, none of the 4 BCoV positive animals manifested any clinical signs of the infection at the time of sample collection. Bayesian majority-rule cladogram comparing partial and full length BCoV RdRp genes obtained in the study to data from the GenBank revealed that the sequences obtained from this study formed one large monophyletic group with those from different species and countries. The goat sequences were similar to each other and clustered within the same clade. No major variations were thus observed between our isolates and those from elsewhere.

**Conclusions:**

Given that Ghana predominantly practices the extensive and semi-intensive systems of animal rearing, our study highlights the potential for spillover of BCoV to small ruminants in settings with mixed husbandry and limited separation between species.

## Background

Bovine Coronavirus (BCoV) belongs to the genus *Betacoronavirus* within the *Coronaviridae* family [1–3]. It is an enveloped, single-stranded and positive-sense RNA virus with a genome size of 32 kb, encoding five main structural proteins: the nucleocapsid, the hemagglutinin esterase, the membrane, the spike (S), and the envelope proteins [4].

BCoV has been implicated in severe diarrhea in neonatal calves, winter dysentery in adult cattle and has been associated with respiratory infections in calves and feedlot cattle [5, 6]. Transmission is primarily through respiratory or fecal-oral routes [7], infecting the respiratory (nasal, tracheal, and lung) and intestinal (villi and crypts of the ileum and colon) epithelial cells [8]. Although infection with BCoV has a low mortality, it generally presents with a high morbidity among cattle of all ages [9]. Outbreaks characteristically occur in autumn and winter, when the virus is most active [10, 11]. Substantial economic toll can be exerted by BCoV infection when a large herd are infected, resulting in drastic reduction in milk yield [12, 13].

In many developing countries where different animals live in close proximity, there is the possibility of interspecies transmission of zoonotic diseases. A few studies have reported the detection of BCoV in small ruminants [14–18].

Despite the health and economic significance of BCoV in livestock, only limited studies have been conducted to evaluate BCoV infection in livestock in Ghana to date. We have recently reported a high seroprevalence, cross-species infection and serological determinants of BCoV in cattle, sheep and goats in Ghana [19]. In this study, we employed a molecular-based detection method to investigate the presence of BCoVs in rectal samples of cattle, sheep and goats from four major regions in Ghana. We also characterized, for the first time, the occurrence and the molecular phylogeny of the BCoVs within the selected regions.

## Results

### The prevalence of BCoV

**Fig. 1:**
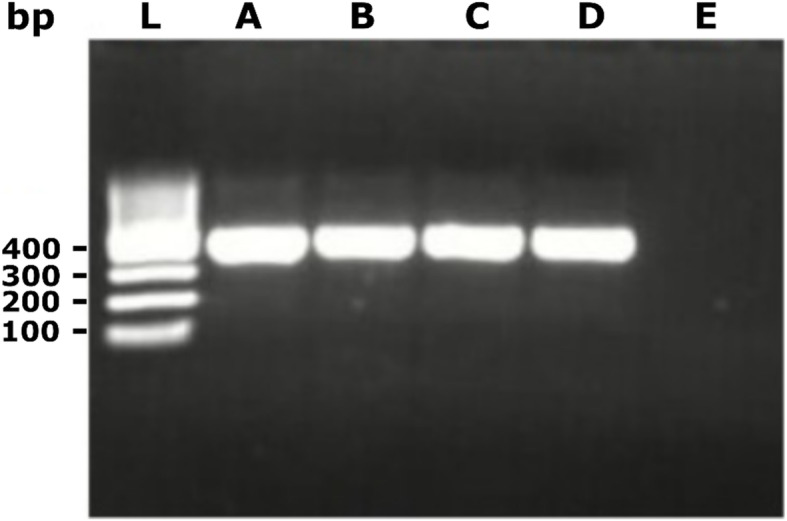
Gel image of the Hemi-nested PCR products used for sequencing. L: 100 bp ladder; A: goat 1; B: goat 2; C: goat 3; D: cattle 1; E: negative control. All products were at the expected size of 404 bp

**Fig. 2:**
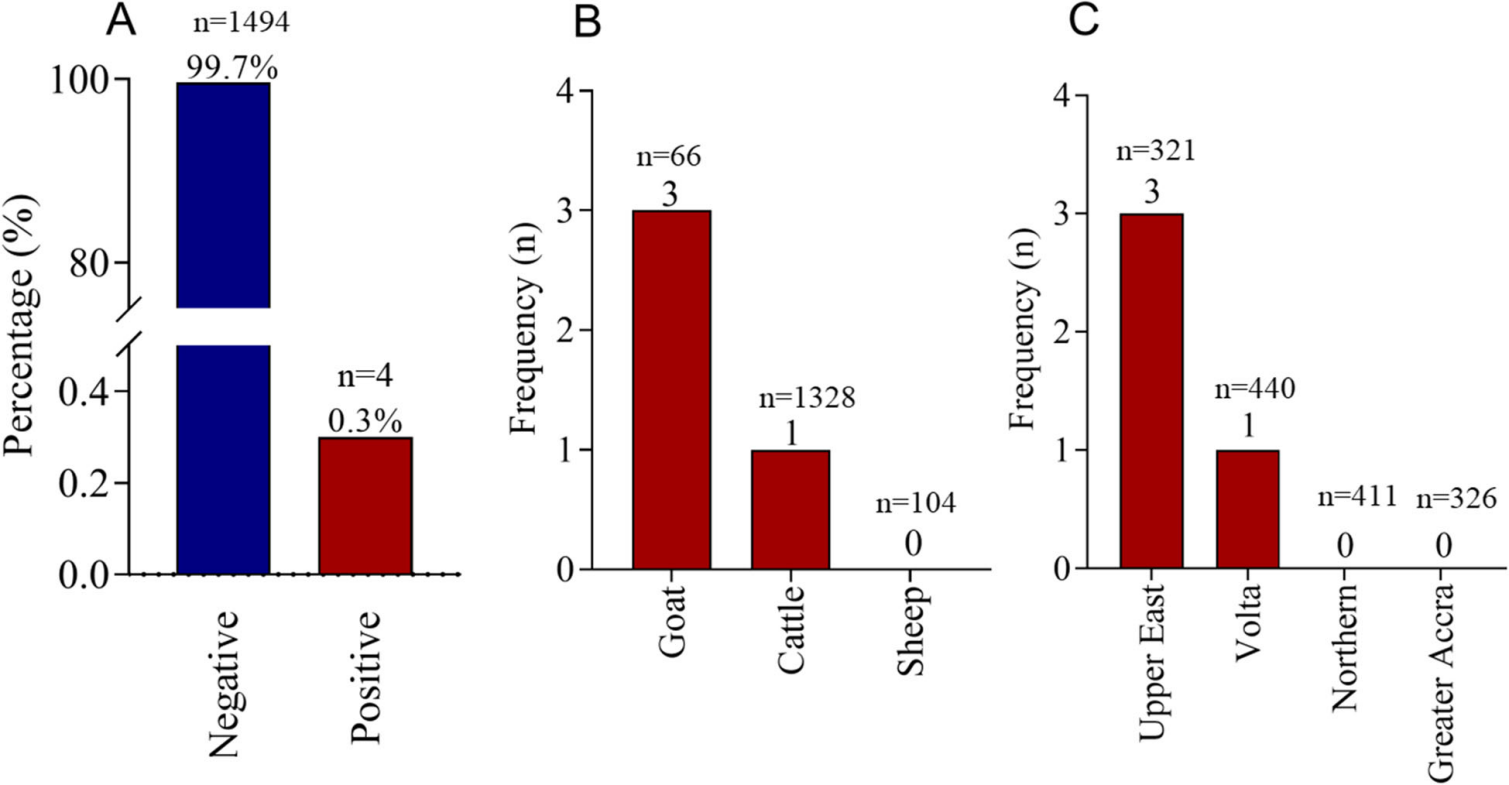
The prevalence of BCoV. **a** Overall prevalence of BCoV among the 1498 animals. **b** Number of BCoV positive cases per type of animal. **c** Number of BCoV positive cases per region. ***n*** represented the total number of animals in each group

Molecular detection of BCoV was based on the use of the RNA-dependent RNA polymerase (RdRp) gene. The prevalence of BCoV in the entire animal population was 0.3% (4/1,498) (Figs. 1 and 2a). Out of the 4 positive cases, 3 were goats whereas 1 was cattle (Fig. 2b). Based on stratification by the sampling regions, the three BCoV positive goats were from the Upper East region while the BCoV positive cattle was from the Volta region (Fig. 2c).

### Sequencing of the RdRp region

**Fig. 3:**
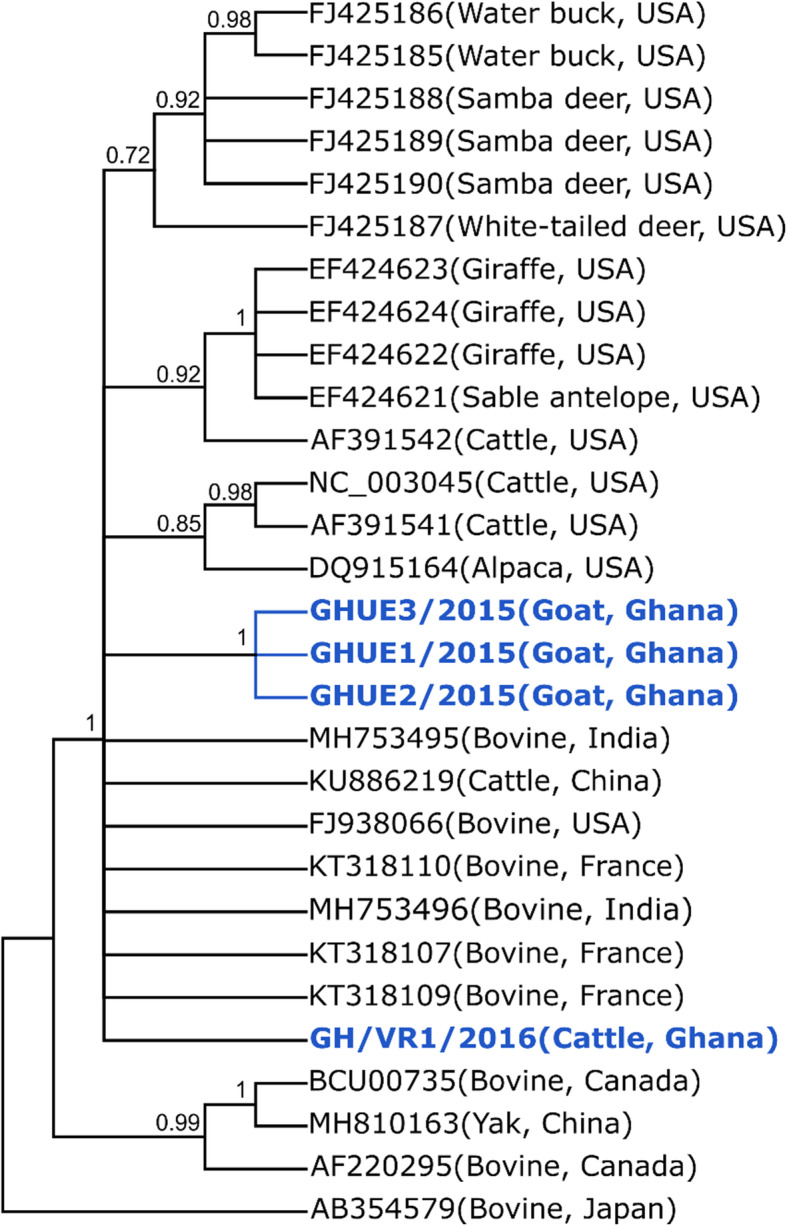
Bayesian majority-rule cladogram comparing partial and full length BCoV RdRp obtained in the study to those from different species and countries

Sequencing was performed on the partial RdRp region of the four bovine coronavirus isolates. The three goat sequences were identical to each other and were 98.56% similar to the cattle sequence based on percentage sequence identity. There were three nucleotide substitutions between the goat sequences in comparison to the cattle sequence. Comparing all sequences to a reference bovine coronavirus sequence from GenBank (Accession number: NC_003045) showed nucleotide substitutions at positions 15281C > T and 153,511 T > A for the goat samples, and at 15,272 G > T, 15,291 C > T, 15,311 T > C and 15,446 G > A for the cattle sample. Figure 3 shows a cladogram constructed for all four bovine coronaviruses identified in goats and cattle. Sequences obtained from this study formed one large monophyletic group with those from different species and countries. The goat sequences were similar to each other and clustered within the same clade. Hence, no major variations were observed with our isolates and those from elsewhere. All sequences were submitted to NCBI and were assigned accession numbers MT711466 to MT711469.

## Discussion

In a prior study, we reported a high seroprevalence, cross-species infection and serological determinants of BCoV in cattle, sheep and goats in Ghana [19]. We found the seroprevalence to be higher in cattle, followed by goats and sheep. For cattle, seroprevalence was significantly higher on larger farms and in the northern region of Ghana, where the climate is relatively dry. In this study, we sought to investigate the presence of BCoVs in rectal samples of cattle, sheep and goats from four major regions in Ghana using molecular-based detection method. We also aimed to characterize the molecular phylogeny of the BCoVs within the selected regions.

This is the first report on molecular detection and phylogenetic analysis of BCoV infection in both cattle and small ruminants in Ghana. The molecular prevalence of BCoV was 0.3%. Among the positive cases, 0.2% were samples obtained from the Upper East region whereas 0.1% was from the Volta region. Greater Accra and Northern regions did not record positive cases. Strikingly, upon stratification by animal type, three out of the four BCoV positive cases were goats whereas one was cattle. No case was recorded in sheep.

The prevalence of BCoV in this study is lower compared to previous studies. A study by Lojkić et al. in Croatia (2015) found 82 of the 101 analyzed fecal and three nasal samples (81%) to be positive for BCoV by RT-PCR [9]. Another study by Kumar et al. in India reported 9.38% (15/160) prevalence of BCoV in cattle [20]. The higher prevalence in these studies could be due to the fact that they considered only cattle that were symptomatic, presenting with diarrhea. This potentiates the likelihood of obtaining samples that are positive for BCoV compared to large-scale randomized screening used in this study. Studies by Lathrop et al. [21], Cho et al. [22], and Hasoksuz et al. [23] also reported a similarly high prevalence of BCoV in the USA. Other factors that could account for the lower prevalence in this study are the disparities in geographical location and timing of sampling relative to viral shedding. Of note, BCoV persists longer in lower temperatures and therefore is able to remain active in the environment all year round in temperate regions compared to the tropical region. Additionally, differences in animal management systems between developing countries such as Ghana and the developed countries could account for the differences in prevalence rates.

Smith et al. indicated that farm management systems have a significant influence on BCoV infection rate [24]. In Ghana, the extensive and semi-intensive systems are largely practiced in cattle, sheep, and goats rearing. On the other hand, the feedlot system of management is a commonly practiced system in most developed countries. This system is a variant of intensive farming practice where animals are confined throughout the year. Such relatively close confinement could enhance the transmissibility of BCoV infection within herds or flocks, thus increasing the detection rate of the virus. Furthermore, BCoV infection is generally self-limiting [25]. Viral shedding is transient and is known to last for only about 9 days. Given that sampling of this study was randomized, with almost all the animals included being asymptomatic, it is possible that sampling was done at a time when viral shedding had terminated.

In Ghana and many developing countries, different animals live in proximity to one another. There is thus the need to investigate cross-species infection because the close and sustained interaction between different animals poses an increased risk of spillover of communicable diseases between animals. However, globally, there are only a few studies evaluating the prevalence of BCoV in non-cattle livestock [14–18]. The presence of BCoV in three goats out of the four BCoV positive cases, thus, indicates a possible active infection and provides update information of spillover of BCoV from cattle to small ruminants in Ghana. It is instructive to state that in Ghana, mixed farming (livestock) is what is largely practiced by farmers and this predispose animals to cross-species infections. The higher prevalence of BCoV observed in goats compared to cattle is interesting, especially when the number of cattle samples were higher than goat samples and the fact that BCoV show tropism for cattle. This can be explained by the role of proximity in the spread of BCoV. All the three BCoV positive goats were from one farm in the Upper East region (Northern Ghana) while the BCoV positive cattle was from the Volta region (Southern Ghana). Additionally, these farms had mixed species. It is important to note that none of the animals which tested positive for BCoV showed clinical signs at the time of sample collection.

Comparison of the nucleotide sequences obtained in this study with sequence data contained in the National Center for Biotechnology Information (NCBI) GenBank revealed no major differences. Three of the four bovine coronavirus isolated were found in goats and one from cattle. Sequences obtained from the goats (Ghana Goat 1–3) were similar to each other and clustered within the same clade. Likewise, the sequence obtained from cattle (Ghana cattle) was also similar to those from other countries. Hence, no major variations were observed with our isolates and those from elsewhere. However, this finding corroborates our previous deposition of the possible interspecies transmission of BCoV from cattle and wildlife to small ruminants [19].

Of note, given the relatively higher prevalence of BcoV in goats compared to cattle, it will be important for future studies to consider sequence analysis of regions other than the highly conserved RdRp. This would be beneficial for phylogenetic analysis of BCoV strains found in goats.

## Conclusions

Given that Ghana predominantly practice the extensive and semi-intensive systems of animal rearing, our study highlights the potential for spillover of BCoV to small ruminants in settings with mixed husbandry and limited separation between species.

## Methods

### Study design/area and data collection

This was a cross-sectional study conducted from January 2015 to December 2018 in Ghana. Animals included in this study consist of 66 goats, 104 sheep and 1,328 cattle from five different districts in Ghana. A simple two-stage cluster sampling technique was used as previously described [19]. The districts included were North Tongu in Volta, Bongo District in the Upper East, Ada West in Greater Accra and Savelugu and Wale wale in Northern region. The number of farms and animal species from the four regions are shown in Table 1.

**Table 1:** Number of farms and animal species from the four regions

Regions	Upper East			Northern			Greater Accra			Volta			Total
Animals	F1	F2	F3	F1	F2	F3	F1	F2	F3	F1	F2	F3	
Cattle	68	100	100	103	131	127	100	100	100	157	139	103	1328
Sheep	21	0	0	12	13	6	10	3	6	12	14	7	104
Goat	11	9	12	8	4	7	2	1	4	0	5	3	66
Grand Total	100	109	112	123	148	140	112	104	110	169	158	113	1,498

F: farms; The three livestock from various households close to each other were pooled together to form a single farm

### Sample collection, processing and analysis

Rectal swabs were collected from each animal using sterile swab sticks. The swabs were placed in pre-labeled cryotubes (SARSTADT, Nümbrecht, Germany) containing a viral RNA stabilization solution, RNAlater (Applied Biosystems, Foster City, CA, USA). Samples were then transported to the Kumasi Centre for Collaborative Research (KCCR) for storage at -70 °C prior to laboratory analysis.

Data and sample collection was done on the owners’ farms and in their presence, after which animals were released back to the owners. Less than 10% of all the animals sampled showed clinical signs such as diarrhea and respiratory distress except for high temperature which occurred in > 1000 of the animals [19].

### Processing of rectal swabs for RNA extraction

Prior to RNA extraction and subsequent laboratory analysis, rectal samples were thawed at room temperature and vortexed. This was followed by centrifugation at 4000 g for 1–2 min before aliquoting 140 µl into new sterile tubes.

### Testing of faecal swabs from cattle, sheep, and goats using RT-PCR

#### Viral RNA extraction

Viral RNA was extracted from all 1,498 samples using the spin protocol of the QIAamp Viral RNA Mini kit (Qiagen, Hilden, Germany) following the manufacturer’s instruction. The RNA was eluted in 100 µl of buffer AVE (pre-warmed at 80^o^C). The eluted RNA was stored at -20^o^C until they were tested for BCoV using real-time polymerase chain reaction (RT-PCR).

### PCR product generation

Detection of bovine CoV RNA was carried out using One-Step RT-PCR Kit (Qiagen, Hilden, Germany) and HCoV-OC43 primers. The following thermal protocol was used: reverse transcription at 50 °C for 30 min, Taq polymerase inactivation at 95 °C for 15 min, 45 cycles of 95 °C for 15 secs, and 60 °C for 30 secs. Amplification results were acquired at 60 °C. The total volume of the Qiagen one-step Master Mix was 25 µl per sample comprised of RNAse-free water (10.5 µl); Onestep 5 x buffer (5 µl); dNTP (1 µl); Forward primer (1 µl); Reverse primer (1 µl); OC43 probe (0.5 µl); enzyme mix (1 µl) and the 5 µl of the RNA template. The oligonucleotide sequences of the forward and reverse primers used for the amplification were CGATGAGGCTATTCCGACTAGGT and CCTTCCTGAGCCTTCAATATAGTAACC respectively, and the probe sequence used was TCCGCCTGGCACGGTACTCCCT as previously described [26]. All RNA extracts were tested for host DNA to determine successful nucleic acid purification prior to use in PCR testing.

### Purification of DNA products for sequencing

All bovine coronavirus positive samples were confirmed by means of 1-step reverse transcription-heminested PCR, using primers CoV2A-F (CTTATGGGTTGGGATTATCC) and CoV2A-R (TAATAACAGACAACGCCATCATC) for the first round and the inner primers CoV2A-Rnest a (CCATCATCACTCAGAATCATCA) and CoV2A-Rnest b (CCATCATCAGAAAGAATCATCA) as previously described [27]. This generated a 404-base pair amplicon from the RNA-dependent RNA polymerase (RdRp) gene (Additional file **1**: Original uncroppped gel image). The detection and sequence analysis were based on the RdRp region because it is a highly conserved region which will facilate broad detection capability across the Betacoronavirus clade 2a.

The PCR products were then prepared for Sanger sequencing by mixing 5 µL of the product with 2 µL of ExoSAP-IT™ (Thermo Fisher, MA, USA) and incubated at 37^o^C for 15 minutes. After this, the mixture was incubated at 80^o^C for 15 minutes and then stored at 4^o^C until use. A volume of 3 µL of each of these cleaned products was then pipetted into 2 tubes and 6 µL of RNAse-free water added to each tube. A volume of 1 µL of the forward primer was added to one tube and the same volume of nested reverse primers to the other tube to give a 10 µL total volume per tube. Sanger sequencing was done by Seqlab GmbH, GÖttingen Germany.

All obtained sequences from Seqlab were compared to sequences deposited on GenBank via the BLAST Algorithm and were aligned together with reference sequences from the Genbank. A cladogram was constructed using Bayesian inference which compared the partial BCoV RdRp sequence obtained from this study to those from different species and countries.

### Statistical Analysis

Categorical data were presented as frequencies (percentages). Analysis of sequence data was done using the online BLAST tool (http://blast.ncbi.nlm.nih.gov/Blast.cgi) to identify homologous strains. Construction of the phylogenetic tree was done by Bayesian inference using MrBayes [28] plugin in Geneious Prime 2019 (http://www.geneious.com). Graphical presentation was performed using GraphPad Prism 7 version 7.04 (GraphPad Software, Inc., La Jolla, California USA).

## Data Availability

The datasets generated and/or analysed during the current study are accessible from Figshare repository: 10.6084/m9.figshare.12830405.v1. The sequence data are available at the National Center for Biotechnology Information (NCBI) (Genbank accession numbers: MT711466-9).

## Abbreviations

BCoV: bovine coronavirus
RdRp: RNA-dependent RNA polymerase
RT-PCR: reverse transcriptase polymerase chain reaction
NCBI: National Center for Biotechnology Information
